# Detection-Aware Delayed Multi-Sensor Fusion for UAV GNSS Spoofing Mitigation: A Factor-Graph-Based Framework

**DOI:** 10.3390/s26216716

**Published:** 2026-10-23

**Authors:** Yupeng Zhang, Yankun Xu, Chengxin Wen, Chenchen Liu, Shaoxun Liu, Qingyang Lu, Peng Fu

**Affiliations:** 1 Endogenous Security Research Center, Purple Mountain Laboratories, Nanjing 211111, China; zhangyupeng@pmlabs.com.cn (Y.Z.);; 2 Beijing Institute of Astronautical Systems Engineering, Beijing 100076, China

**Keywords:** GNSS spoofing detection, multi-sensor fusion, factor graph, UAV navigation, software-in-the-loop simulation, CUSUM detector, spoofing mitigation

## Abstract

GNSS spoofing can progressively corrupt unmanned aerial vehicle (UAV) navigation when manipulated position solutions are fused before integrity assessment. Existing detection methods often use dead-reckoning references such as visual–inertial odometry (VIO) without fully accounting for accumulated drift or GNSS-dependent global alignment. This paper presents a closed-loop software-in-the-loop (SITL) evaluation platform and a detection-aware delayed multi-sensor fusion framework for UAV GNSS spoofing mitigation. The platform couples state estimation, flight control, vehicle dynamics, and sensor feedback to enable Monte Carlo evaluation under realistic navigation–control interactions. A cumulative sum (CUSUM) detector monitors consistency between GNSS and drift-compensated VIO, while recent GNSS factors are quarantined and incorporated into a factor graph only after integrity screening. Experiments quantify false-alarm rate, detection probability, and latency across detector settings and spoofing deviation rates. Onboard implementation and controlled injection experiments further assess real-time feasibility and hardware transferability. Under the evaluated sensor conditions and gradual-drift attack model, a 10–15 s window yielded detection probabilities exceeding 96% for attacks with a deviation rate of 0.5 m/s, with an approximately 3% false-alarm rate. These findings demonstrate the framework’s potential for spoofing mitigation and the platform’s utility for closed-loop evaluation.

## 1. Introduction

Unmanned aerial vehicles (UAVs) are increasingly deployed in logistics, infrastructure inspection, precision agriculture, disaster response, environmental monitoring, and other safety-critical applications [1,2]. These missions require the vehicle to estimate its position and motion state with low latency and sufficient reliability. Global Navigation Satellite Systems (GNSS) remain the most widely used source of global absolute positioning for outdoor autonomous systems, owing to their broad coverage and all-weather availability under nominal satellite visibility [3,4]. In practical UAV navigation systems, GNSS is therefore commonly integrated with inertial measurement units (IMUs), cameras, LiDARs, and other exteroceptive sensors to improve the temporal resolution, observability, and robustness of state estimation [5,6]. GNSS provides a long-term global reference, whereas inertial and visual sensors supply high-rate local motion constraints. Without periodic absolute-position corrections, however, dead-reckoning systems suffer from bias accumulation and cannot generally maintain reliable long-term positioning accuracy [6,7].

Despite its widespread use, GNSS cannot be regarded as an intrinsically trustworthy navigation source [8,9]. Satellite signals are extremely weak by the time they reach the Earth’s surface, and most civil services lack adequate authentication mechanisms [10]. An adversary can exploit these characteristics to transmit counterfeit signals or manipulate GNSS measurements in a manner sufficiently consistent with the receiver’s expected observations [11]. In contrast to jamming, which primarily denies or degrades GNSS availability, spoofing can gradually displace the estimated position of a UAV while leaving the receiver apparently operational [12]. For a UAV, such covert trajectory manipulation can corrupt guidance commands, flight-control actions, waypoint tracking, airspace compliance, collision-avoidance decisions, and mission execution [13,14,15].

Countermeasures against GNSS spoofing are generally divided into two classes: signal-level detection and information-level detection [8,9]. Signal-level approaches exploit the physical or propagation characteristics of GNSS signals to identify anomalies, and include signal-quality monitoring, multi-antenna direction finding, and signal-authentication schemes [10]. These methods, however, typically require hardware modifications to the GNSS receiver and remain vulnerable to sophisticated spoofing that mimics authentic signal characteristics. In contrast, information-level methods leverage auxiliary measurements from onboard sensors, such as IMUs and vision systems, to provide independent cross-validation. By validating GNSS outputs against complementary sources of motion information, such approaches offer an effective means of GNSS integrity monitoring without altering the receiver front end.

Within this paradigm, an IMU provides relative-motion information and can expose deception by checking the internal consistency of the navigation solution [16]. Nevertheless, the significant noise inherent in commercial-grade IMUs precludes their use for standalone navigation [17], so an IMU alone can hardly distinguish a slowly varying spoofing attack from the unbounded growth of its own inertial errors. In GPS-denied environments, exteroceptive sensors such as cameras and LiDARs are therefore commonly employed to mitigate drift [6,18]. Visual–inertial odometry (VIO) is a representative fusion scheme that exploits the complementary characteristics of cameras and IMUs: cameras provide geometric constraints from tracked scene features, while IMUs supply high-rate angular-velocity and specific-force measurements for state propagation between consecutive image frames [19,20,21]. This complementarity enables VIO to estimate short-term relative motion with bounded drift, and the same reasoning applies to LiDAR–inertial odometry (LIO) and LiDAR–inertial–visual odometry (LIVO) [22,23]. These advantages have motivated the use of VIO and LIO as auxiliary references for GNSS spoofing detection [24,25,26,27].

Two coupled deficiencies nevertheless limit the credibility and effectiveness of existing spoofing-detection studies. The first concerns evaluation fidelity: detectors are typically assessed by superimposing artificial errors on recorded GNSS data or by replaying manipulated navigation solutions along a prescribed trajectory. The KITTI dataset, for instance, has been modified to emulate spoofing scenarios [24,25], and in other studies spoofing-free data were first collected and various attacks were subsequently simulated offline [26,27]. Such open-loop settings omit the causal chain of an actual attack. In real flight, spoofed position feedback alters the estimated state, which in turn affects the flight-control commands and the vehicle dynamics, and the resulting deviation of the true trajectory then propagates into the IMU, camera, and other sensor observations. Consequently, the interplay among the attacker, the estimator, and the controller—which ultimately determines whether an attack is detected and how the vehicle responds—is never exercised. The second deficiency concerns the temporal coupling between real-time positioning and integrity assessment. To meet real-time navigation demands, common position fusion frameworks may incorporate GNSS measurements before their integrity has been fully established. Spoofed measurements can therefore bias the estimated state and subsequent predictions, allowing the estimator to track the spoofed trajectory and suppressing the residuals used for integrity monitoring [13,14,15]. Such a failure mode is particularly pronounced for slowly varying or induced spoofing, where a gradually increasing position bias can be absorbed into the estimated state without producing an abrupt, readily detectable inconsistency.

This paper addresses both deficiencies. We first establish a closed-loop software-in-the-loop (SITL) simulation platform that emulates realistic UAV ego-motion under GNSS spoofing, so that spoofing detection can be evaluated subject to the same positioning–control feedback that governs real flights. A key insight revealed by this platform is that a spoofed UAV exhibits dynamic closed-loop behavior that fixed-trajectory injection tests cannot reproduce: the flight controller reacts to falsified position feedback and actively adjusts the trajectory, thereby altering subsequent sensor observations and vehicle dynamics.

On this basis, we propose a detection-aware delayed multi-sensor fusion framework for small UAVs using factor-graph optimization (FGO). The framework does not immediately insert every GNSS solution into the factor graph. Instead, incoming GNSS factors are buffered and admitted only through a detection-aware delayed-fusion procedure. This design ensures the real-time of navigation requirements, and also limits the ability of spoofing to steer the fused position estimate or contaminate the state estimator before an alarm is raised. Evaluation across varying spoofing severities and fusion delays demonstrates that the proposed framework can effectively prevent slowly varying spoofing errors from immediately steering the fused position estimate or contaminating the detector’s internal state.

The estimator is further implemented on a lightweight airborne computing platform, confirming that the proposed method satisfies the stringent latency and memory requirements of onboard deployment. Controlled GNSS injection experiments further demonstrate its practicality for trustworthy UAV positioning in low-altitude economic operations.

The main contributions of this paper are summarized as follows:

1. **A closed-loop SITL evaluation platform.** We establish an SITL framework that explicitly couples spoofed GNSS measurements with state estimation, flight-control feedback, vehicle dynamics, and subsequent sensor observations, enabling spoofing detection to be assessed under realistic positioning–control loops rather than through static data injection.
2. **A detection-aware delayed multi-sensor fusion positioning framework.** We integrate a loosely coupled factor-graph estimator with a cross-consistency detector. GNSS solutions are temporarily quarantined and fused only after integrity verification, which mitigates the risk of spoofing-induced state contamination prior to detection.
3. **A systematic analysis of the trade-off between fusion delay and detection performance.** We quantify the cost–benefit balance of the proposed framework across varying spoofing severities and fusion delays, and show that it prevents slowly varying spoofing errors from contaminating the fused navigation state.
4. **A real-time onboard implementation and controlled injection validation.** We demonstrate computational feasibility on a lightweight aerial platform and confirm experimental consistency between the SITL simulation and the behavior of the implemented navigation pipeline.

The remainder of this paper is organized as follows. Section 2 formulates the problem and details the proposed resilient FGO-based multi-sensor fusion framework. Section 3 describes the closed-loop SITL evaluation platform and the simulation studies on detection performance across various detector settings and spoofing deviation rates. Section 4 presents a validation on the lightweight hardware platform and the controlled GNSS injection experiments. Section 5 discusses parameter selection, the inherent difficulty of detecting stealthy spoofing, and the limitations of the present study, and Section 6 concludes the paper.

## 2. Methodology

### 2.1. System Overview

**Figure 1:**
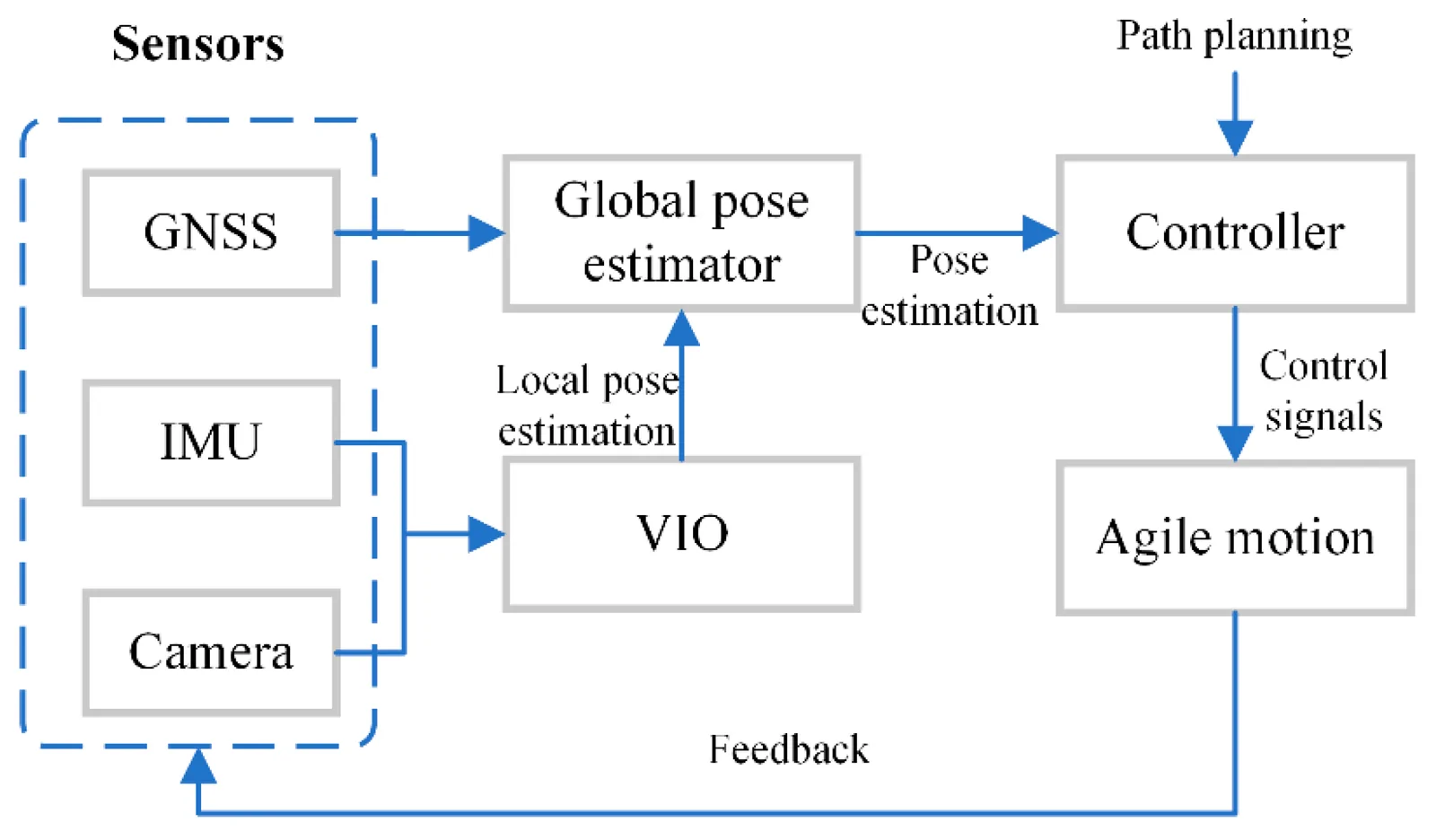
Overview of the closed-loop positioning–control architecture of lightweight UAVs.

This work focuses on the positioning and navigation requirements of lightweight small UAVs. To reproduce realistic UAV ego-motion under GNSS spoofing, a closed-loop positioning—control architecture is adopted, as shown in Figure 1. The system integrates a carefully selected multi-source sensor suite with a loosely coupled state estimator to achieve robust global localization, while a path planner and a controller govern the UAV motion according to the localization solution. The agile motion of the UAV then changes its position in the environment, which in turn affects the onboard sensor measurements.

The onboard sensors are deliberately chosen to strike a balance among positioning accuracy, payload weight, and computational efficiency, which are critical constraints for small UAVs. Specifically, a GNSS receiver provides absolute global positioning information, an IMU delivers high-rate angular velocity and linear acceleration measurements, and stereo cameras capture visual features for environmental perception. It should be noted that the sensor suite is not limited to those mentioned above, and may also include additional effective sensors.

As for the multi-sensor fusion positioning, existing fusion strategies fall into two categories: loosely coupled and tightly coupled architectures. In a loosely coupled architecture, a GNSS receiver first produces an independent position or velocity solution, which is then fused with the VIO output in a secondary estimator [28]. Tightly coupled fusion, by contrast, directly integrates raw GNSS measurements (pseudorange, Doppler shift, and carrier-phase observables) with visual and inertial constraints within a unified estimation framework [29,30]. By retaining more of the original measurement geometry, tightly coupled estimation can improve observability and accuracy, particularly when only a limited number of GNSS measurements are available. However, its performance depends strongly on calibration, measurement quality, observability, and the consistency of the visual and inertial constraints. A further concern is that tightly coupled systems are more sensitive to measurement degradation, including those induced by spoofing, since degraded raw observations are directly fed into a unified optimization framework where state variables from all sensors are mathematically coupled [31]. Consequently, loosely coupled methods remain preferable for resource-constrained platforms due to their lower complexity and modularity [28]. These considerations motivate the loosely coupled architecture adopted in this work.

Specifically, the IMU and cameras are tightly coupled within a VIO module to produce a local pose estimate. This local estimate is subsequently fused with the GNSS measurement in a Global pose estimator, yielding a robust Pose estimation that compensates for the drift inherent in pure VIO.

On the planning and control side, a Path Planning module generates the desired trajectory or mission objectives, which serve as reference commands to the Controller. By leveraging the globally fused pose estimation as real-time state feedback, the Controller computes appropriate Control signals to drive the Agile motion execution module, thereby governing the physical dynamics and maneuvering of the UAV. The resulting platform motion is continuously measured by the onboard sensors, feeding back into the perception layer and closing the loop. This integrated closed-loop framework ensures accurate state estimation, responsive trajectory tracking, and stable agile maneuvering for lightweight UAV applications.

### 2.2. Local Pose Estimation

VIO is a well-established technology, and many impressive algorithms have been developed. Among these, filtering-based and optimization-based methods are the most commonly used. Filtering-based methods maintain a compact state and update it recursively as new measurements arrive. Usually, the landmark constraints are excluded from the filter state through the null-space projection [19,32]. This design provides bounded computational complexity and limited memory usage, but incur information loss that limits accuracy. Optimization-based methods based on sliding-window bundle adjustment [20] or incremental smoothing and mapping (iSAM) [33,34] have also achieved real-time performance on resource-constrained platforms and generally attain better accuracy. Such methods can jointly refine poses of the vehicle and landmarks, but their computational cost increases with the number of observations.

SchurVINS revisits the relationship between filtering and bundle-adjustment formulations from a Schur-complement perspective [21]. Rather than eliminating landmark constraints solely through the conventional null-space projection adopted in filter-based estimators, SchurVINS leverages the Schur-complement to separate the pose-related and landmark-related components. This hybrid architecture preserves joint constraints between poses and landmarks without explicit manipulation of the high-dimensional full state vector, thereby attaining real-time performance comparable to filtering methods while approaching the accuracy of optimization-based counterparts. In this paper, we adopt SchurVINS as the local pose estimator. For completeness, we briefly introduce its state representation here; further details can be found in the original work [21].

The output of the local VIO is represented in the local frame ℱ*_L_*. The state vector is defined as

(1) xIL=qITL,pITL,vITL,baT,bgTT,

where qIL, pIL and vIL are the orientation, position and velocity with respect to the local reference frame ℱ*_L_*, respectively. ***b**_a_* and ***b**_g_* individually represent the biases of the linear acceleration and angular velocity measured by the IMU device. In the state vector, the orientation is represented as a quaternion, whereas in the covariance matrix the orientation-related part is parameterized in Euler angles. The corresponding EKF error-state of *^L^**x**_I_* is defined as

(2) x˜IL=θ˜ITL,p˜ITL,v˜ITL,b˜aT,b˜gTT,

where θ˜IL represents the error-state of qIL.

In SchurVINS, the basic state xIL is augmented to improve estimation accuracy and consistency by explicitly modeling multi-keyframe constraints. The augmentation state is defined as

(3) X=xITL, xA1TL, xA2TL, …, xANTLT,

where xAiL=qI,iTL,pI,iTLT is the augmented historical pose state. The corresponding covariance ***P*** is partitioned matrix as

(4) $$P=\left[\begin{array}{cc} P_{\mathrm{II}} & P_{\mathrm{IA}}\\ P_{\mathrm{IA}}^{T} & P_{\mathrm{AA}} \end{array}\right],$$

where ***P**_II_* and ***P**_AA_* are the covariance of basic state and augmented state, respectively. ***P**_IA_* is the covariance between basic state and augmented state.

### 2.3. Detection-Aware Delayed Multi-Sensor Fusion Framework Based on FGO

**Figure 2:**
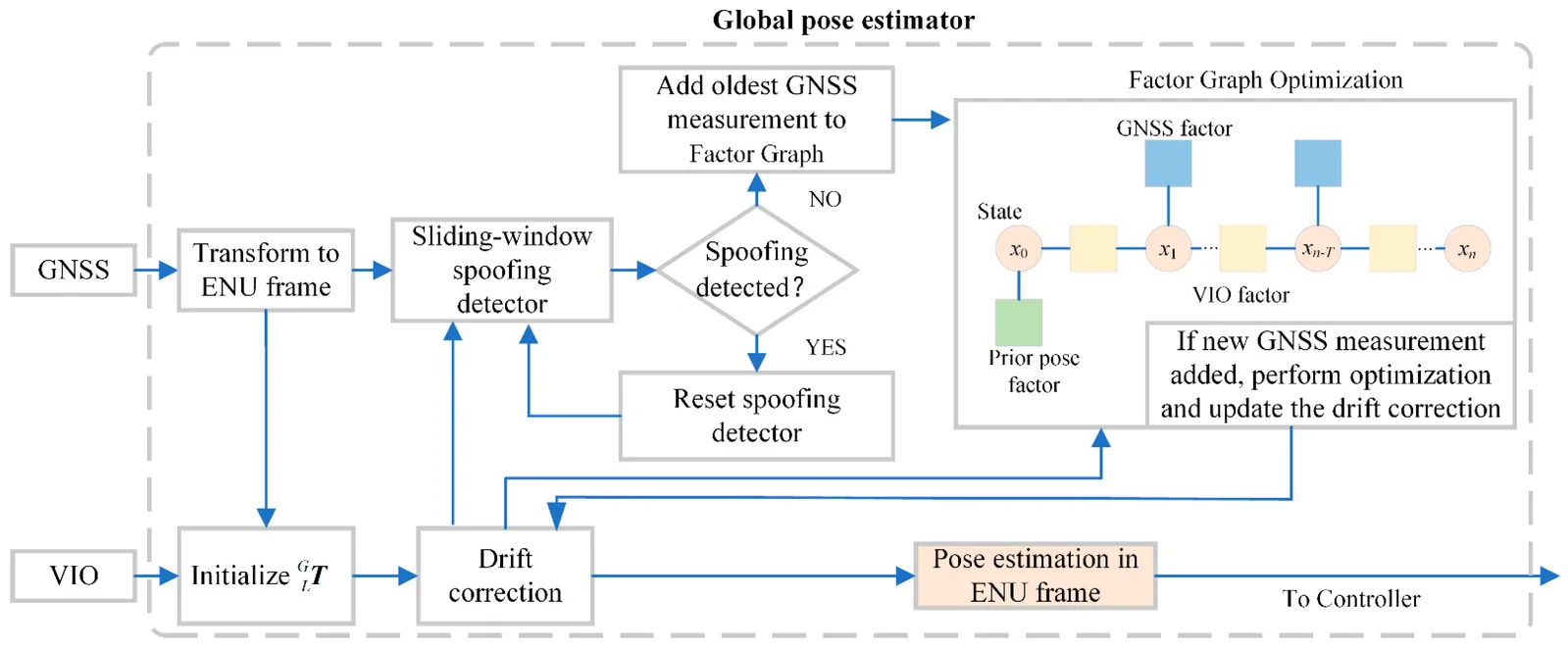
Architecture of the proposed resilient multi-sensor fusion positioning framework.

Compared with recursive EKF filtering, factor-graph optimization computes a maximum a posteriori (MAP) estimation over multiple poses and sensor measurements, enabling re-linearization, robust constraints, and consistent marginalization. This generally leads to more accurate state estimates and more consistent covariance estimates. Therefore, we propose a resilient multi-sensor fusion positioning framework based on FGO as the global pose estimator, as shown in Figure 2. At the core of the architecture is a factor-graph optimization module that maintains a temporally ordered set of state nodes constrained by prior, VIO relative-pose, and GNSS absolute-position factors. GNSS data are first transformed into the East-North-Up (ENU) frame, which serves as the global frame ℱ*_G_*, and a sliding-window spoofing detector continuously monitors GNSS integrity to identify anomalous or spoofing signals. Upon detection of a spoofing attack, the system resets and reinitializes the monitoring window; otherwise, the validated GNSS measurement is added to the factor graph as a GNSS factor. Whenever a new validated GNSS measurement is incorporated, the global estimator performs MAP estimation over the active window and updates the drift-correction parameters used to compensate for VIO drift. In parallel, VIO outputs are aligned to global frame ℱ*_G_* through initialization and drift-compensation modules, and the resulting motion prior is also used to assist GNSS spoofing detection.

In the factor graph, each node is defined as xG=qTG,pTGT, which contains orientation and position in global frame ℱ*_G_*. The MAP estimation problem of the global estimator can be defined as [35]

(5) $$\begin{array}{cc} X^{*} & =\arg\underset{X}{\max}\prod_{k=0}^{n}\;\prod_{s\in S}p(X|z_{s,k})\\ & =\arg\underset{X}{\max}\prod_{k=0}^{n}\;\prod_{s\in S}p(z_{s,k,}|X) \end{array}$$

where χ=x0G, x1G, x2G, …,  xnG are global poses of all nodes. ***z**_s_*_,*k*_ denotes the measurements acquired from different sensors, while ***S*** denotes the set of sensors. In the inference of Equation (5), the prior *p*(*χ*) over the states is assumed to be uniform. It is worth noting that, in practice, a prior pose factor (as shown in Figure 2) is attached to the initial state *x*_0_ solely to anchor the reference frame (i.e., to fix the gauge freedom), according to the corresponding GNSS and VIO measurements with large uncertainties. *p*(***z**_s_*_,*k*_|*χ*) denotes the uncertainty of measurements ***z**_s_*_,*k*_, which is usually assumed to follow a Gaussian distribution:

(6) $$p(z_{s,k}|X)\propto \exp\left(-\frac{1}{2}{\left\|h\left(X\right)-z_{s,k}\right\|}_{\Sigma_{s,k}}^{2}\right),$$

where *h*(·) represents the measurement model of the sensor. ${\left\|e\right\|}_{\Sigma }^{2}=e^{T}\Sigma^{-1}e$ is defined as the squared Mahalanobis distance with covariance matrix ***Σ***. Consequently, the MAP estimation problem can be equivalently formulated as

(7) $$X^{*}=\arg\underset{X}{\min}\sum_{k=0}^{n}\sum_{s\in S}{\left\|h\left(X\right)-z_{s,k}\right\|}_{\Sigma_{s,k}}^{2}.$$

GNSS typically provides measurements of longitude, latitude and altitude (*φ*, *λ*, *h*) in geodetic GNSS coordinates. The measurements (*φ*, *λ*, *h*) must be converted to the ENU frame with respect to a selected reference point (*φ*_0_, *λ*_0_*, h*_0_). The subsequent GNSS measurements are denoted as pGNSSG. Thus, the GNSS absolute-position factors can be represented as

(8) hxkG−zGNSS,k=pkG−pGNSS,kG.

Since VIO pose estimates are expressed in the local frame ℱ*_L_*, they provide the relative transformation between successive states. Therefore, the VIO factor is formulated based on the relative pose between two consecutive pose states.

(9) hxk−1G,xkG−zVIO,k=qk−1−1GqkGqk−1−1G(pkG−pk−1G)⊖qk−1−1LqkLqk−1−1L(pkL−pk−1L),

where ⊖ denotes the minus operation on the relative states. The corresponding variance of the relative pose estimation is derived as [36]

(10) $$\Sigma_{k-1,k}=\left[-\begin{array}{cc} A & I \end{array}\right]\left[\begin{array}{cc} P_{k-1} & P_{k-1,k}\\ P_{k-1,k}^{T} & P \end{array}\right]{\left[\begin{array}{cc} -A & I \end{array}\right]}^{T},$$

where ***I*** denotes the identity matrix and ***P**_k_* represents the adjoint matrix of ***z**_VIO_*

(11) $$A=\left[\begin{array}{cc} R_{k-1,k}\cdot P_{k-1} & 0\\ {\left\lfloor t_{k-1,k}\right\rfloor }_{\times }R_{k-1,k} & R_{k-1,k} \end{array}\right],$$

where ***R**_k_*_−1,*k*_ is the rotation matrix corresponding to qk−1−1LqkL. The skew-symmetric matrix associated with the relative translation vector tk−1,k=qk−1−1L(pkL−pk−1L) is denoted as

(12) $${\left\lfloor t\right\rfloor }_{\times }=\left[\begin{array}{ccc} 0 & -t_{z} & t_{y}\\ t_{z} & 0 & -t_{x}\\ -t_{y} & t_{x} & 0 \end{array}\right].$$

To obtain the optimal global states for the factor graph, the iSAM2 (incremental smoothing and mapping) algorithm implemented in the GTSAM library is employed. iSAM2 maintains an adaptive Bayes tree that confines re-linearization and re-elimination to only the trajectory segments affected by new measurements or loop closures [34,35]. In this way, the full pose graph is preserved while the computational cost remains bounded, thereby enabling incremental MAP estimation in a computationally efficient manner.

### 2.4. Spoofing Detection Scheme

To validate the GNSS measurements, the local pose estimate must first be transformed into the global frame. Since both frames are gravity-aligned, the relative rotation RLG between them can be parameterized by a single yaw angle during initialization.

(13) RLG=cosφ−sinφ0sinφcosφ0001,

where *φ* is the relative yaw angle between the two frames. The transformation between the local frame and the global frame can be expressed as TLG=RLG | tLG, where tLG denotes the relative translation between the origins of the two frames.

To compensate for the inherent drift of VIO, a drift-correction transformation is estimated from the fused global poses. The most recent fused states are used to estimate a slowly time-varying correction transformation ***T**_c_* = [***R**_c_*|***t**_c_*]. The translational correction is calculated using a weighted Euclidean average and the rotational correction is derived using the Markley quaternion averaging method.

(14) M=∑i=k−N+1kω¯i ⋅qiG ⋅qLG⋅qiL−1,

(15) tc=∑i=k−N+1kω¯i ⋅piG−Rc,i⋅  RLG⋅piL,

where qLG is the unit quaternion corresponding to RLG and ***R**_c_*_,*i*_ is the rotation matrix corresponding to ***q**_c_*_,*i*_,

(16) Rc,i←qc,i=qiG ⋅qLG⋅qiG−1.

The normalized weight is

(17) $${\bar{\omega }}_{i}=\frac{\omega_{j}}{\sum_{j=k-N+1}^{k}\omega_{j}},$$

*ω_j_* = *tr*(***P**_II_*_,*j*_) ⋅ exp(*j* − *k*), *j* ∈ [*k* − *N* + 1, *k*], (18)

where *tr*(·) denotes the trace of a matrix.

Then, the averaged quaternion is obtained as the unit eigenvector corresponding to the largest eigenvalue of ***M***,

(19) $$R_{c}\leftarrow q_{c}=\arg\underset{x}{\max}\left(x^{T}\cdot M\cdot x\right),\;s.t.\;{\left\|x\right\|}_{2}=1.$$

Therefore, the corrected VIO position in the global frame at the current time is calculated as

(20) pVIO,kG=Tc⋅TLG⊙pkL,

where ⊙ denotes the operator that applies the rotation transform to a position vector.

An uncertainty-normalized innovation statistic is defined

(21) dk=pVIO,kG−pGNSS,kGΣr,k2,

where ***Σ**_r_*_,*k*_ ≈ ***Σ**_GNSS_*_,*k*_ + ***Σ**_VIO_*_,*k*_ and ***Σ**_VIO_*_,*k*_ approximates the uncertainty of the drift-compensated VIO position pVIO,kG, using the position uncertainty reported by the FGO estimator as a reference. This approximation is used for residual normalization and does not represent a complete uncertainty propagation through the drift-correction transformation.

Under the nominal hypothesis that the GNSS measurement is consistent with the VIO prediction, the statistic approximately follows a chi-square distribution with three degrees of freedom. To detect persistent or gradually increasing GNSS inconsistencies, the normalized innovation statistic is accumulated using a one-sided CUSUM detector:

*s_k_* = max{0, *s_k_* _− 1_ + *d_k_* − *κ*}, *s*_0_ = 0, (22)

where *κ* is the reference value controlling the trade-off between noise rejection and detection sensitivity. The detector raises an anomaly alarm when *s_k_* ≥ *γ*, where *γ* is the detection threshold. The sliding-window GNSS spoofing detector is summarized in Algorithm 1.
**Algorithm 1.** Sliding-window GNSS spoofing detector**Input:** GNSS measurements pkG, ***Σ**_GNSS_*_,*k*_; aligned VIO measurements pVIO,kG, ***Σ**_VIO_*_,*k*_; time *t_k_*; time window length *t_w_*; parameters *κ* and *γ*.
**Output:** spoofing alert flag, spoofing alarm time *t*_det_; refined spoofing detection time $t_{\det}^{\prime }$.
1:    **Initialize:** *t*_det_ = 0, $t_{\det}^{\prime }=0$

2:    Calculate *d_k_* according to Equation (21)
3:    Update *s_k_* according to Equation (22)
4:    **if**
*s_k_* < *γ* **then**
5:         Add Add pkG to the back of candidate sequence ***Ω***
6:         **while *Ω*** is not empty **do**:
7:             Take the front element piG in ***Ω***
8:             **if** *t_k_* − *t_i_* ≥ *t_w_* **then**
9:                 Add GNSS measurement piG related factor to FG
10:                Remove piG from ***Ω***
11:            **else**
12:                break
13:            **end if**
14:        **end while**
15:        **if** new GNSS factor added **then**
16:            Perform the FGO
17:           Update VIO drift-correction parameters according to Equations (14)–(19)
18:        **end if**
19:    **else**
20:        Set spoofing alert flag
21:        Update *t*_det_ = *t_k_*
22:        **while Ω** is not empty **do**:
23:            Take the back element piG in ***Ω***
24:            Calculate *d_i_* according to Equation (21)
25:            **if** *d_i_* ≥ *κ*
**then**
26:               Update *s_k_* by *s_k_* = *s_k_* − (*d_i_* − *κ*)
27:               Remove piG from ***Ω***
28:            **else**
29:               Update $t_{\det}^{\prime }=t_{i}$

30:               Update *s_k_* by *s_k_* = max(*s_k_*, *γ*)
31:               Remove piG from ***Ω***
32:               break
33:            **end if**
34:        **end while**
35:    **end if**

The GNSS and VIO measurements are synchronized via interpolation and the VIO position is drift-compensated and transformed to the global frame using Equation (20). Then, the aligned GNSS and VIO measurements are passed to the sliding-window GNSS spoofing detector as summarized in Algorithm 1. The detector maintains a candidate sequence that buffers incoming GNSS measurements, while an uncertainty-normalized innovation statistic *d_k_* between each GNSS measurement and the drift-compensated VIO estimate is computed and accumulated into a detection statistic *s_k_* following a one-sided CUSUM detector. When the detection statistic *s_k_* is below the alert threshold *γ*, the candidate sequence ***Ω*** is examined in chronological order, starting from the oldest measurement. A buffered GNSS measurement acquired at time *t_i_* is eligible for incorporation into the factor graph only when its age satisfies *t_k_* − *t_i_* ≥ *t_w_*, where *t_k_* is the current processing time and *t_w_* is the prescribed time window length. Measurements with *t_k_* − *t_i_* < *t_w_* remain buffered and are not yet incorporated. If the oldest measurement does not satisfy the age condition, the examination stops because all subsequent measurements are younger. When one or more GNSS factors are added, factor-graph optimization is performed, followed by an update of the VIO drift-correction parameters.

When *s_k_* reaches or exceeds *γ*, a spoofing alert is raised and the spoofing alarm time *t*_det_ is recorded. The detector then traces back through its most recent elements, removing the measurements whose residuals exceed *κ* and subtracting their contributions from *s_k_*, until the first consistent measurement is reached. Its timestamp is retained as the refined detection time $t_{\det}^{\prime }$, and the statistic *s_k_* is reset accordingly. In this manner, integrity monitoring is tightly coupled with sensor fusion: validated measurements reinforce the global solution and recalibrate the VIO drift, whereas suspected measurements are isolated before they can propagate into the state estimate.

It is worth noting that the statistic *s_k_* is clamped as *s_k_* = max(*s_k_*, *γ*) after the traceback rather than being reset to zero. This operation implements a hysteresis mechanism: the contributions of confirmed spoofed measurements are removed from *s_k_*, but the detector remains in the alert state until subsequent measurements are sufficiently consistent to drive *s_k_* below *γ*. Such an asymmetric trigger-and-release strategy prevents the detector from oscillating between the normal and alert states.

## 3. Simulation Platform and Simulation Analysis

### 3.1. Overview of the Simulation Platform

**Figure 3:**
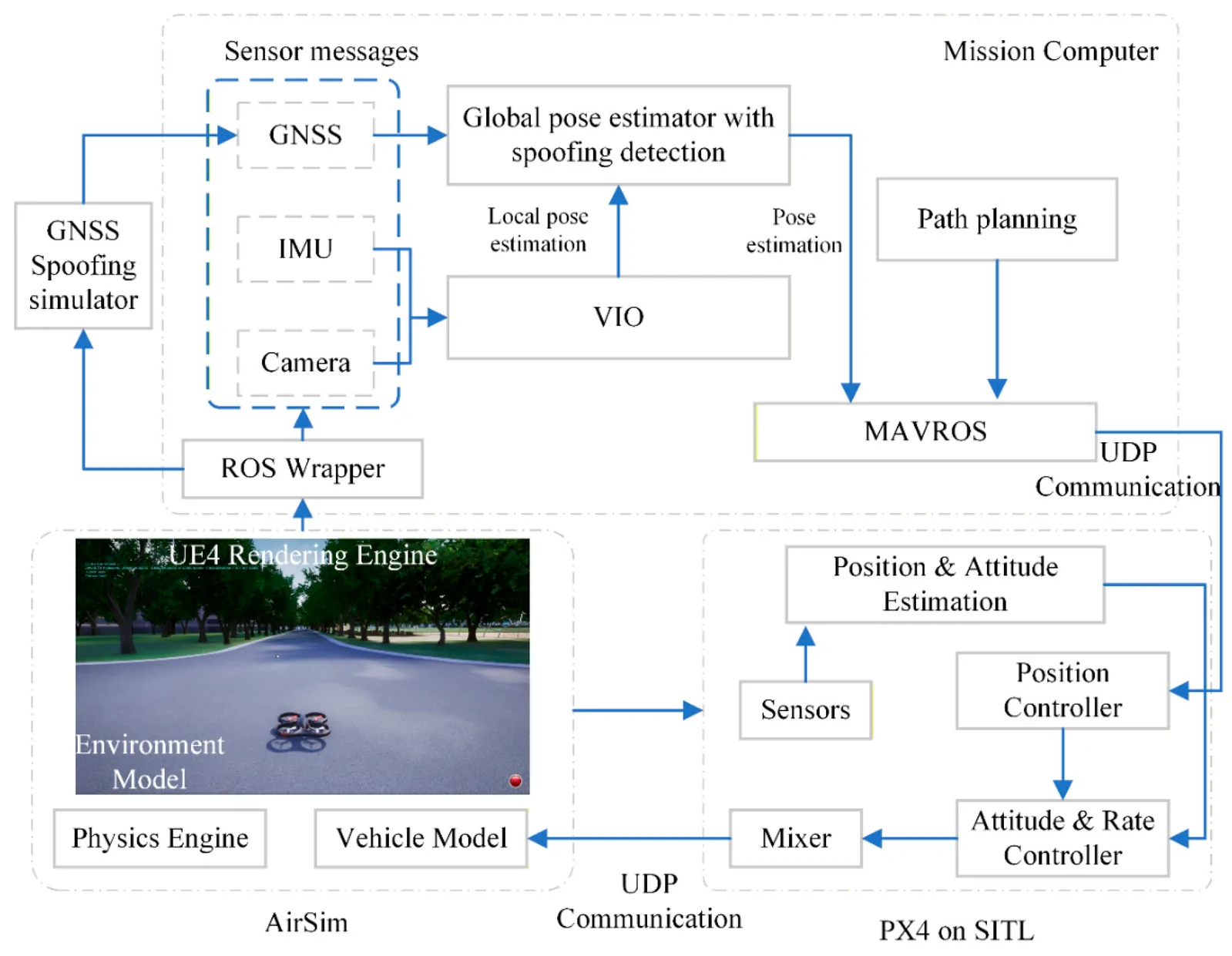
Architecture of the closed-loop SITL evaluation platform.

A closed-loop SITL evaluation platform is established to assess the spoofing detection performance under realistic positioning–control loops, as shown in Figure 3. The platform consists of three main components: the environment and sensor simulator based on the AirSim photorealistic rendering engine (v1.8.1) [37], the SITL flight-control simulator based on the PX4 autopilot (v1.13.1) [38], and the mission-computer simulator hosting the pose estimation and autonomy algorithms. AirSim renders synthetic stereo images and reproduces the visual appearance of the environment under configurable lighting and weather conditions; the PX4 SITL module provides a high-fidelity flight dynamics model and a realistic attitude/position control loop; the mission-computer simulator executes the pose estimation algorithm together with a routine path-planning module. All components are integrated through the Robot Operating System (ROS) middleware, enabling closed-loop data exchange among simulation, estimation, and control.

In AirSim, the UE4 Rendering Engine generates a photorealistic environment model, while the physics engine simulates vehicle dynamics through the vehicle model. Simulated sensor data are published via the ROS Wrapper as sensor messages. A VIO frontend produces local pose estimation, while the global pose estimator with spoofing detection fuses GNSS and VIO measurements to output a drift-free global Pose estimation. Concurrently, a path planning module generates mission trajectories. Both the pose estimation and the planned path are forwarded to MAVROS, which bridges the ROS ecosystem and the PX4 autopilot via UDP Communication.

Within the PX4 SITL domain, the flight control stack receives high-level commands through MAVROS and executes the low-level control loop. Internal Sensors feed into the Position & Attitude Estimation module, whose output is used by the Position Controller to track the commanded trajectory. The Position Controller then drives the Attitude & Rate Controller, which computes actuator commands that are dispatched to the Mixer. The Mixer converts these into individual motor commands, which are transmitted back to the Vehicle Model in AirSim via UDP Communication, thereby closing the entire simulation loop.

A GNSS spoofing simulator was developed and integrated into the data pipeline. It takes the true GNSS positioning from AirSim as input and injects malicious or anomalous GNSS signals in place of the authentic ones. We use a gradual-drift attack in which the spoofed position departs from the true trajectory at a controlled rate, representing an induced spoofing scenario.

(23) $$z_{\mathrm{GNSS}}^{\left(s\right)}\left(t\right)=z_{\mathrm{GNSS}}\left(t\right)+\alpha \cdot \left(t-t_{\mathrm{start}}\right)+\varepsilon \left(t\right),$$

where *α* denotes the deviation rate, *t_start_* is the spoofing start time, and *ε* denotes the GNSS position noise.

### 3.2. Simulation Analysis

The simulation was conducted in the MSBuild2018 (Soccer Field) environment [39], which emulates a UAV performing a security patrol mission over a campus-like area. The simulation was configured with the key parameter settings listed in Table 1. These parameters were selected based on an analysis of the noise characteristics of sensors commonly employed in lightweight UAVs [40,41].

**Figure 4:**
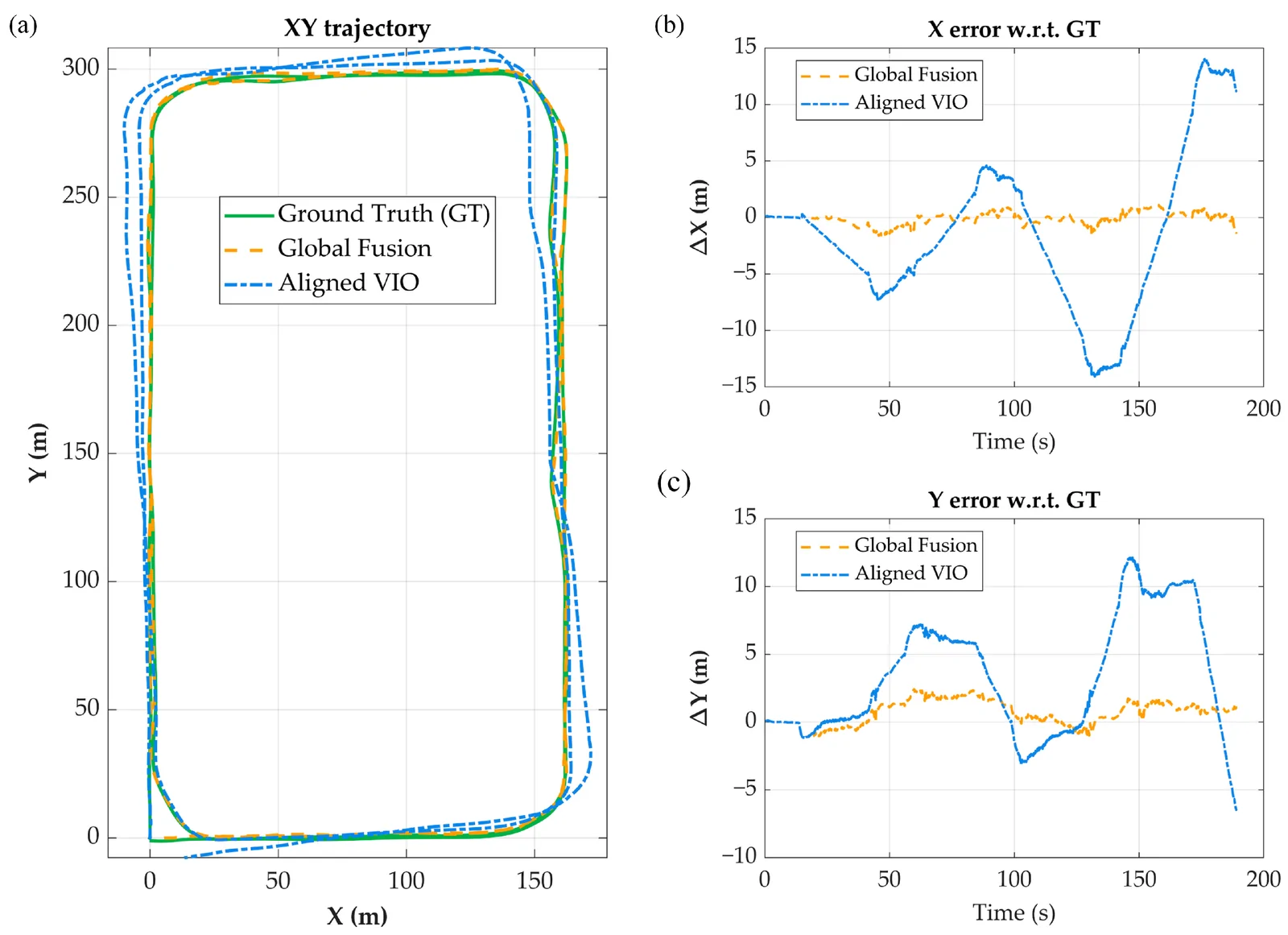
Simulation results in the AirSim MSBuild2018 environment. (**a**) Comparison of the UAV flight trajectories, including Ground Truth (GT), Global Fusion, and Aligned VIO. (**b**) Positioning error along the *X*-axis with respect to GT. (**c**) Positioning error along the *Y*-axis with respect to GT.

Figure 4a illustrates the flight trajectory of the UAV. The different colors represent the ground truth, the global fusion estimate (with time window length *t_w_* = 10 s), and the aligned VIO estimate, respectively. Figure 4b,c show the corresponding positioning errors along the X and Y axes, respectively.

The global fusion trajectory remains close to the ground truth throughout the rectangular patrol mission, whereas the aligned VIO trajectory progressively diverges. This qualitative observation is supported by the error plots in Figure 4b,c. The fusion estimate generally maintains errors within approximately ±2 m along both axes, while the standalone VIO solution accumulates substantial drift and reaches peak errors exceeding 10 m near 130–150 s. These results demonstrate why periodic global constraints are essential for maintaining long-term navigation accuracy.

To quantify the effect of delayed GNSS admission on nominal navigation accuracy, we evaluate the online global fusion estimation accuracy under attack-free conditions across different time window lengths. For each run, the three-dimensional position root mean square error (RMSE) is computed according to

(24) RMSE=1N∑k=1NpG−pGTG22,

where pGTG is the time-aligned ground truth. All runs share identical evaluation configurations except for the time window length *t_w_*, where *t_w_* = 0 s denotes immediate fusion, i.e., GNSS measurements are incorporated without an intentional buffering period once they pass the detection statistic *s_k_* check. The three-dimensional position RMSE is first computed for each complete attack-free run individually. We then report the mean and standard deviation across runs in Table 2. As can be seen, the buffering configurations that incur a measurable nominal positioning cost.

#### 3.2.1. False Alarm Rate Analysis

**Figure 5:**
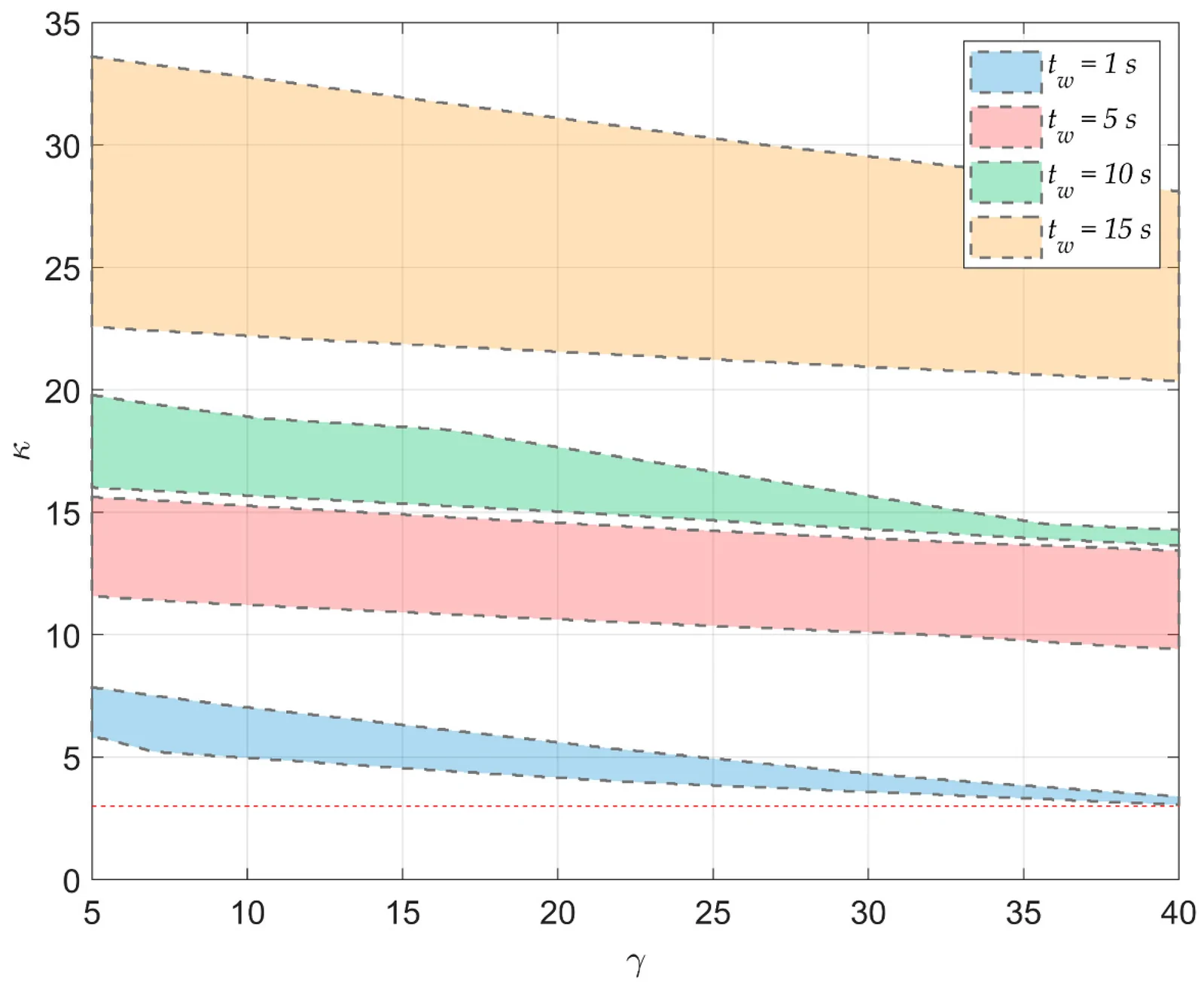
Parameter selection analysis for κ and γ under different time windows length (*t_w_*). The shaded regions indicate the valid parameter space where the FAR is maintained within the target range of 3–5%. The red dashed line represents the expected value of the theoretical chi-square distribution with three degrees of freedom.

Reliable operation requires the false-alarm rate (FAR) to remain within an acceptable range. We therefore performed 100 independent Monte Carlo runs under nominal, non-spoofed conditions. The VIO, GNSS, and global-fusion outputs were recorded, and the detection statistic *d_k_* was computed according to Equation (21). We then evaluated the FAR over the parameter space of the reference parameter κ, decision threshold γ, and window length *t_w_*. The false-alarm rate is evaluated on a per-run basis. Specifically, a run is counted as a false-alarm run if the detector raises at least one spoofing alarm during an otherwise attack-free run. The FAR is then defined as the ratio of false-alarm runs to the total number of attack-free runs. Figure 5 identifies configurations for which the FAR remains within the target range of 3–5%.

Figure 5 depicts the relationship between the threshold parameters κ and γ under various time window settings (*t_w_*). The shaded areas denote the feasible parameter space in which the system FAR remains within the acceptable range of 3–5%, with the upper and lower edges of each band corresponding to the maximum and minimum κ values that satisfy this constraint. Two trends can be observed. First, for a fixed *t_w_*, the feasible range of κ shrinks as γ increases, reflecting the nonlinear coupling between the FAR and the threshold parameters (κ, γ). A small γ renders the FAR less sensitive to κ, making small γ values preferable for robust parameter selection. Second, larger windows (e.g., *t_w_* = 15 s) require substantially higher thresholds than smaller ones (e.g., *t_w_* = 1 s). This is attributable to the progressive drift of the standalone VIO solution over longer durations: as the accumulated drift inflates the nominal detection statistic, a higher threshold becomes necessary to keep the FAR low. Based on these bounds, appropriate (κ, γ) pairs can be selected as listed in Table 3.

To provide an immediate-fusion reference, we include a configuration with *t_w_* = 0 s. The CUSUM detector structure is retained, while the window-specific parameter settings are listed in Table 3. In our simulation tests, this baseline achieves a FAR of no more than 3%, and the results are therefore omitted from Figure 5.

It should be noted that time windows longer than 15 s (e.g., *t_w_* = 20 s and 25 s) lie outside the desired FAR design space and are therefore omitted from Figure 5. As evidenced by the simulations, such prolonged windows elevate the FAR to approximately 10–20% when using the parameters listed in Table 3. This degradation can be attributed to the progressive accumulation of VIO drift over the observation interval, which inflates the nominal detection statistic *d_k_* and thus compromises its reliability. We nevertheless retain these settings in Table 3 to characterize the extended sensitivity–latency trade-off rather than to recommend them for deployment.

#### 3.2.2. Performance Analysis Under Different Spoofing Deviation Rates

To evaluate the spoofing detection capability of the proposed system, a series of Monte Carlo simulations was performed in which the UAV was subjected to GNSS spoofing attacks with varying deviation rates α ∈ {0.5, 1.0, 1.5, 2.0, 3.0} m/s. Here, α characterizes the severity of the attack, i.e., the rate at which the spoofed GNSS positioning progressively deviates from the true trajectory. Each simulated flight lasted approximately 120 s, and the spoofing attack was initiated at a random time between 70 s and 80 s. To ensure statistical reliability, the simulation was independently repeated 100 times, with a new random waiting time generated for each repetition. The detection performance was assessed under seven time window settings *t_w_* ∈ {0, 1, 5, 10, 15, 20, 25} s using two metrics: detection rate and detection time. Detection rate is defined as the proportion of complete attack runs in which the spoofing detector raises a valid spoofing alarm within the prescribed evaluation interval. As for the detection time, two time quantities are distinguished in Algorithm 1. The spoofing alarm time *t*_det_, is the time at which the detector raises a spoofing alarm. The retrospectively refined detection time $t_{\det}^{\prime }$, is obtained subsequently by tracing back through the buffered measurements. Owing to the delayed fusion mechanism, the refined detection time $t_{\det}^{\prime }$ reflects the GNSS measurements that can be excluded from fusion, provided that the spoofing attack is successfully detected. Consistent with the detection rate, we report the detection time as the mean and standard deviation across complete runs. The results are summarized in Table 4.

**Figure 6:**
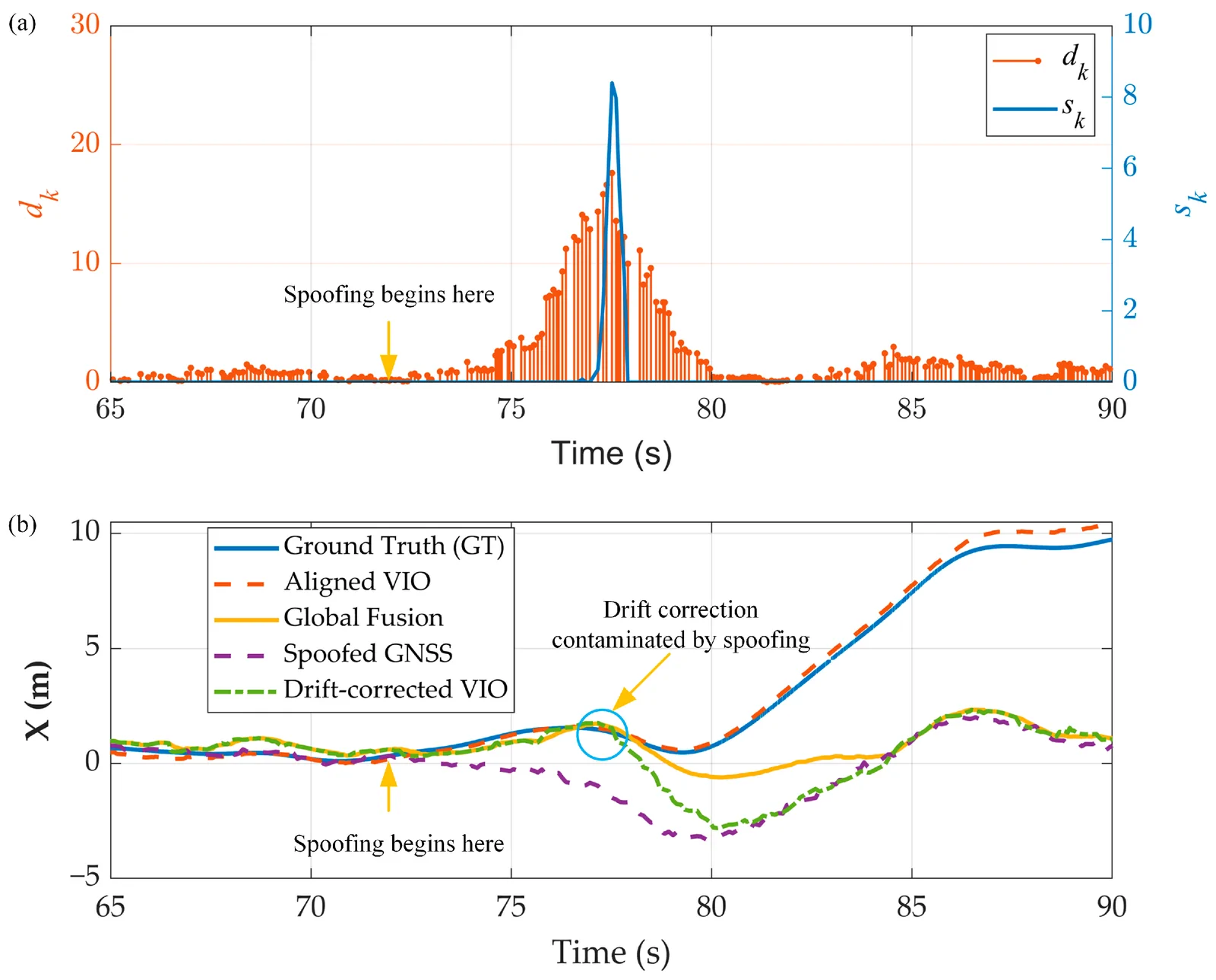
Case study of a slow-deviation-rate spoofing attack (*α* = 0.5 m/s) with a time window length of *t_w_* = 5 s. (**a**) Normalized innovation statistic *d_k_* (left axis) and CUSUM statistic *s_k_* (right axis). (**b**) Position estimates along the *X*-axis. The spoofing attack begins at t ≈ 72 s (yellow arrow); the drift correction is contaminated by the spoofed GNSS solution at t ≈ 77 s (circled region), because no alarm is triggered.

The time window length directly affects the system’s sensitivity to subtle attacks. As shown in Table 4, attacks with slow drift rates (α ≤ 2.0 m/s) are hardly detectable with *t_w_* = 1 s. For *t_w_* = 0 s (the baseline of immediate fusion), detection is even more challenging: no effective detection was achieved in our test scenarios, so no results are reported for this configuration. The underlying mechanism is as follows: a slowly induced GNSS position deviation gradually pulls the fused solution away from the true trajectory, while the VIO drift correction simultaneously attributes the resulting GNSS–VIO discrepancy to its own drift and compensates for it. As a result, the test statistic never crosses the detection threshold, and the stealthy spoofing evades detection. A representative case is illustrated in Figure 6.

As shown in Figure 6a, the normalized innovation statistic *d_k_* remains near the noise floor before the attack onset at *t* ≈ 72 s. Once spoofing begins, the spoofed GNSS solution gradually deviates from the true trajectory, as indicated by the purple dash-dotted line in Figure 6b, and the resulting GNSS–VIO discrepancy is gradually reflected in *d_k_*. At t ≈ 77 s the statistic *s_k_* rises sharply to about 8.5 but remains below the decision threshold γ and thus fails to trigger the spoofing alarm. Following this near-miss, the spoofed measurements are consequently admitted to the global estimator, and the VIO drift-correction mechanism progressively attributes the discrepancy to its own drift and compensates for it. This suppresses further growth of *d_k_*, preventing the CUSUM statistic *s_k_* from reaching the alarm threshold. The example illustrates the central vulnerability of immediate fusion: once the attack influences the correction state, it can reduce the residual that the detector relies upon.

The position traces in Figure 6b reveal the critical consequences of this failed accumulation. During the interval from *t* ≈ 72 s to 77 s, the spoofed GNSS solution (purple dash-dotted line) steadily diverges in the negative direction. Because spoofing detection fails, the global fusion solution (yellow solid line) is pulled away from the ground truth (blue solid line). The drift-corrected VIO, updated using the contaminated global solution, is then gradually drawn toward the spoofed trajectory, which further suppresses subsequent detection attempts. The aligned VIO estimate (green dashed line), which does not receive the contaminated correction, remains closer to the true trajectory over this interval. However, standalone VIO is not a sustainable long-term solution, as it eventually accumulates drift as discussed earlier. Rather, the comparison demonstrates how early fusion can convert a temporary detection failure into a persistent navigation error.

**Figure 7:**
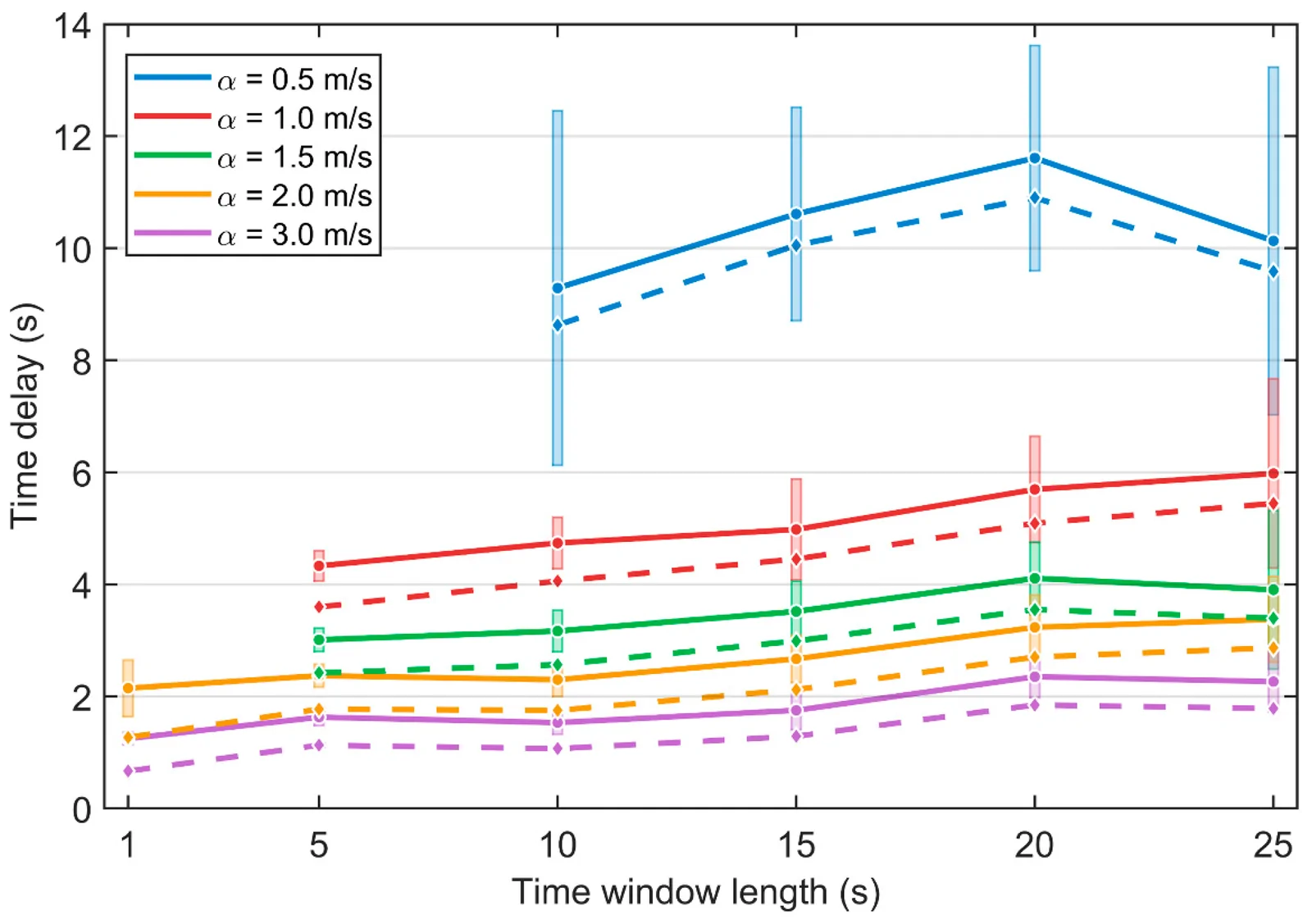
Mean detection time delay for GNSS spoofing attacks as a function of time window length *t_w_* across deviation rates α. Solid lines show the mean spoofing alarm latency (*t*_det_ − *t_start_*), with error bars indicating the standard deviation over 100 independent Monte Carlo runs. Dashed lines show the corresponding mean refined detection time offset. Curves of the same color correspond to the same deviation rate α.

Figure 7 complements Table 4 by showing the mean spoofing alarm latency as a function of *t_w_* for each deviation rate α, with error bars indicating the standard deviation. For a fixed time window length, detection time decreases as α increases because stronger attacks produce evidence more rapidly. On average, the retrospectively refined timestamp precedes the alarm timestamp by approximately 0.6 s. This difference represents the backward temporal extent of the inferred boundary used for buffered-measurement rejection. The delayed-admission mechanism makes this retrospective handling possible for measurements that remain in the buffer when the alarm is raised. For a fixed α, increasing *t_w_* generally increases detection latency because a longer observation interval is required to accumulate sufficient evidence as a higher threshold is adopted to maintain an acceptable FAR. The results therefore reveal a fundamental design trade-off: short time windows respond quickly but are vulnerable to slowly varying attacks, whereas long time windows improve sensitivity at the cost of increased latency.

Taken together, these trends justify selecting *t_w_* ∈ [10,15] s as a balanced operating point. In this range, the system achieves high detection rate for *α* ≥ 0.5 m/s within acceptable time latency.

## 4. Experiments and Results

### 4.1. Overview of the Experimental Setup

The experiment platform comprises a lightweight quadrotor airframe integrated with a flight controller, a companion computer, a multi-constellation GNSS receiver, a visual–inertial sensor, and a laboratory-grade GNSS signal simulator for controlled spoofing injection.

**Figure 8:**
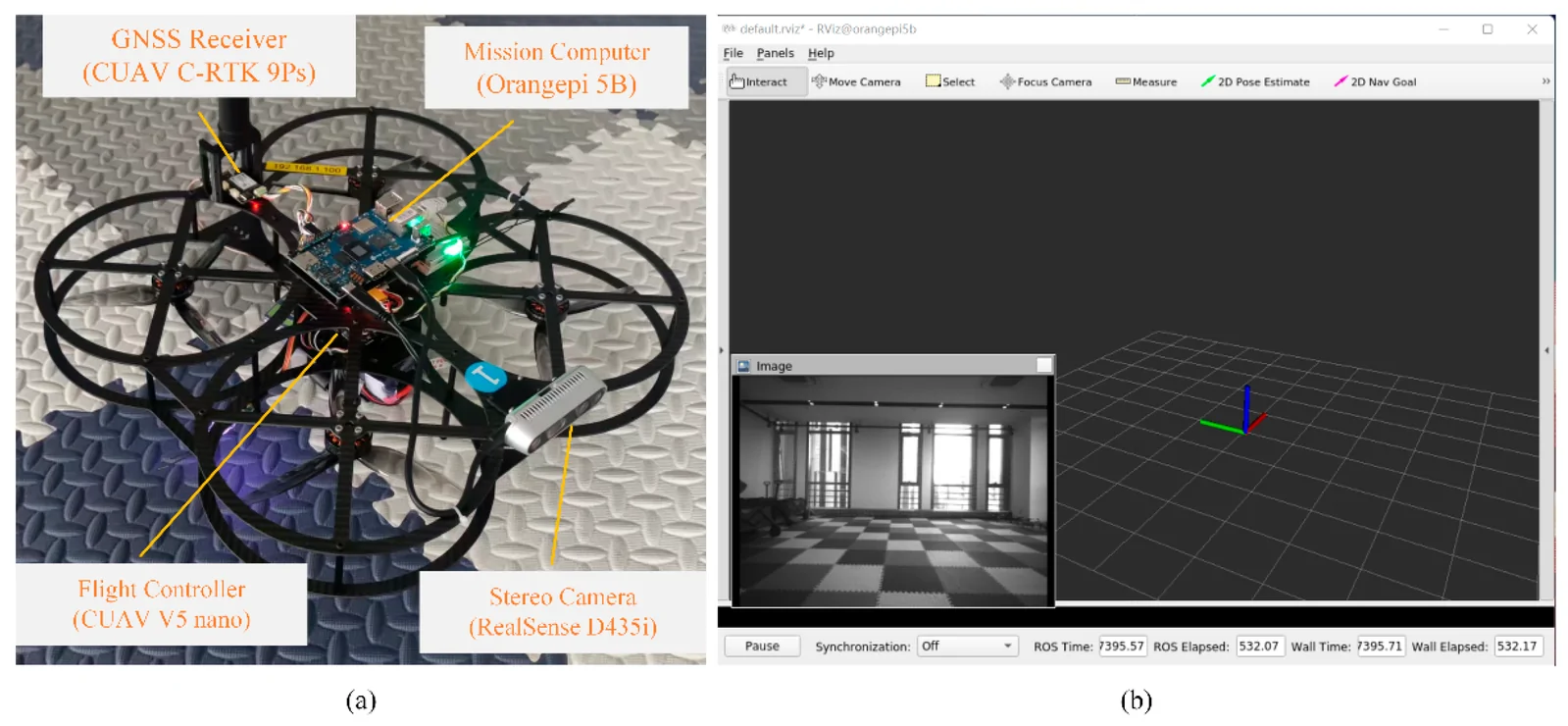
Experimental lightweight UAV platform and its RViz visualization. (**a**) The physical UAV platform. The onboard components are labeled, including a GNSS receiver (CUAV C-RTK 9Ps), a mission computer (OrangePi 5B), a flight controller (CUAV V5 nano), and a stereo camera (RealSense D435i). (**b**) The RViz visualization interface. The bottom-left panel shows the real-time image feed from the stereo camera, and the main panel displays the real-time VIO output as a 3D grid with coordinate axes.

As shown in Figure 8, the experimental UAV platform is a quadrotor equipped with a protective frame. The main onboard components are a RealSense D435i stereo camera (Intel Corporation, California, USA) and a CUAV C-RTK 9Ps GNSS receiver (CUAV Tech Inc., Ltd., Guangzhou, China) for multi-sensor pose estimation, an OrangePi 5B (Orange Pi, Shenzhen, China) mission computer for high-level processing, and a CUAV V5 Nano flight controller (CUAV Tech Inc., Ltd., Guangzhou, China) for low-level control. Figure 8b shows the corresponding RViz visualization, where the bottom-left panel displays the real-time stereo image feed from the camera.

The flight controller is a CUAV V5 nano running the open-source PX4 autopilot firmware (v1.13.1), consistent with the simulation platform. The onboard companion computer is an OrangePi 5B based on the RK3588S system-on-chip, which contains a heterogeneous octa-core CPU (4 × Cortex-A76 at 2.4 GHz and 4 × Cortex-A55 at 1.8 GHz), an ARM Mali-G610 GPU, and an integrated 6-TOPS neural-processing unit. The board runs Ubuntu 20.04 with ROS Noetic and executes the complete localization-to-decision pipeline.

**Figure 9:**
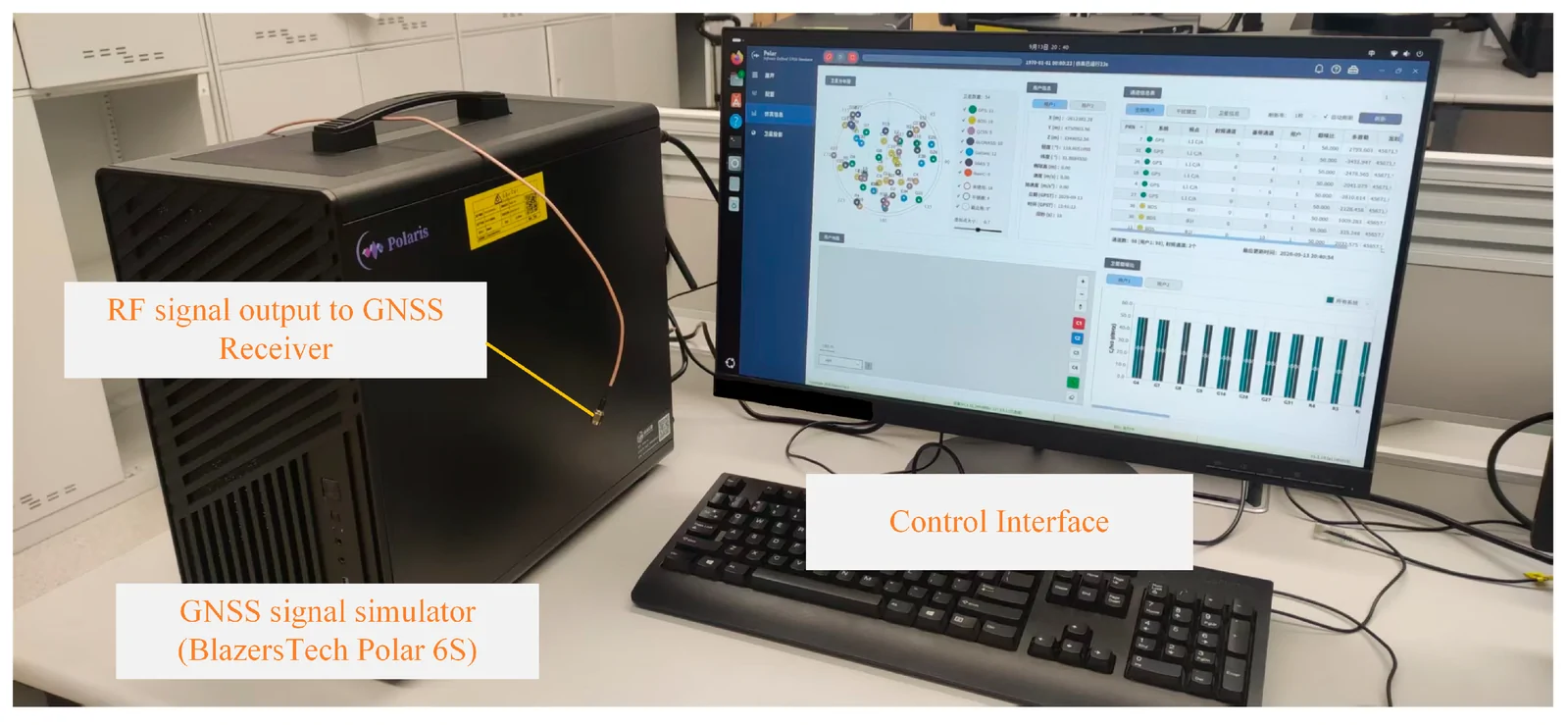
GNSS signal simulator used in the experiment. The GNSS signal simulator generates GNSS RF signals, which are delivered to the UAV’s GNSS receiver via the RF output port. The host computer provides the control interface for constellation configuration and scenario monitoring.

A BlazersTech Polar 6S GNSS signal simulator (BlazersTech, Beijing, China) [42] is employed to generate realistic GNSS radio-frequency (RF) signals under controlled laboratory conditions, as shown in Figure 9. The simulator supports simultaneous multi-constellation, multi-frequency signal generation (GPS, BDS, Galileo, and GLONASS) with configurable navigation data, satellite geometry, and atmospheric delay models. The RF output of the Polar6S is coupled to the antenna input of the UAV’s GNSS receiver via a shielded coaxial cable and an appropriate attenuator chain, ensuring that the simulated (and potentially spoofed) signals dominate the receiver’s tracking loops while preventing unintended RF leakage to the external environment.

The overall data flow on the physical platform mirrors the architecture in Figure 3. All sensor data (GNSS observables, RealSense D435i stereo images, and IMU samples) are published as time-synchronized ROS topics. SchurVINS processes the visual-inertial stream to generate the VIO solution, while the proposed detection-aware delayed fusion module fuses it with the GNSS measurements in real time, yielding the fused navigation solution. Within this module, a CUSUM-based spoofing detector continuously monitors the integrity of the GNSS observations. The fused position is then forwarded to the PX4 flight controller via MAVLink, closing the perception–decision–control loop. This closed-loop integration allows the detection-to-response latency to be evaluated under realistic timing constraints, offering a faithful assessment of the proposed framework’s deployment readiness.

### 4.2. Computational Profiling

**Figure 10:**
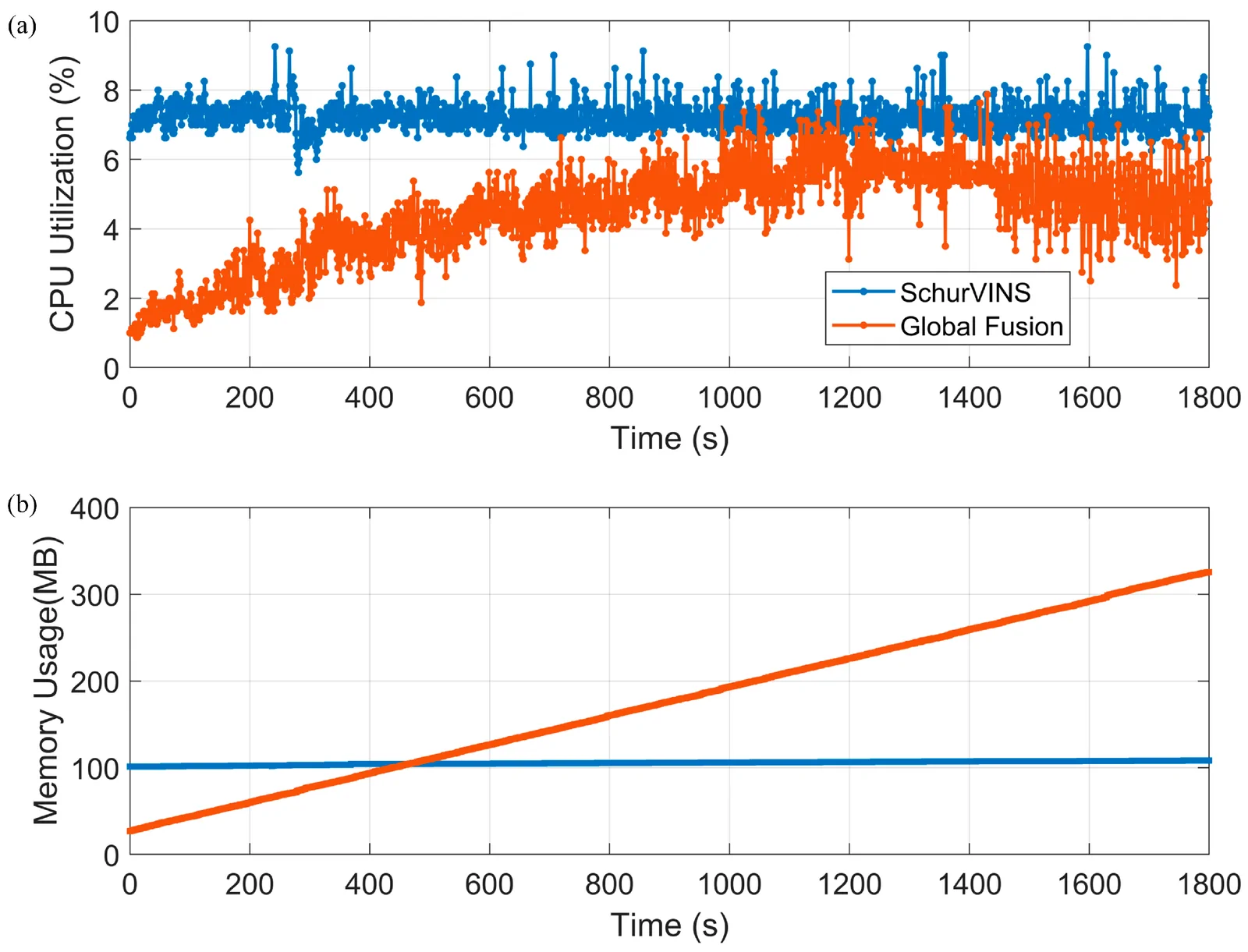
Computational overhead of the proposed framework under nominal conditions (UAV stationary, simulated GNSS RF signals) over a 30 min run. (**a**) CPU utilization. (**b**) Memory usage.

Before conducting the attack scenarios, the computational overhead of the proposed framework was evaluated. With the UAV stationary on a test bench, simulated GNSS RF signals were injected using the Polar6S simulator, and the complete pipeline, including SchurVINS and the global-fusion module, was executed for 30 min. Process-level CPU utilization and memory footprint were recorded for the two individual modules on the onboard companion computer using *pidstat*, which samples the Linux /*proc* filesystem. The results are shown in Figure 10.

As shown in Figure 10a, SchurVINS uses approximately 6–8% CPU, whereas the global-fusion module uses approximately 4–6%, leaving substantial headroom for real-time execution on a resource-constrained mission computer. The memory profiles in Figure 10b show a different trend. SchurVINS maintains an almost constant footprint of approximately 115 MB, while the global-fusion module grows from about 30 MB to 330 MB as the pose graph accumulates VIO and GNSS observations. The global-fusion CPU load increases during the initial graph growth and then remains relatively stable.

For a typical 30 min run, the resource consumption of the global-fusion module remains below 10% CPU and 350 MB memory. These evaluations demonstrate that the proposed framework can run on a representative UAV companion computer without compromising real-time processing. The memory trend indicates that the current implementation is computationally feasible for short and medium-duration missions, but that long-duration operation would benefit from explicit graph marginalization or fixed-lag smoothing.

### 4.3. Spoofing Detection in Controlled GNSS RF Hardware Tests

The UAV platform was placed on an indoor test bench and held stationary. The BlazersTech Polar 6S simulator was configured to first transmit nominal GNSS signals for a 60 s initialization phase, after which a spoofing attack was injected while maintaining while signal continuity was maintained, so that conventional receiver-level spoofing indicators would not be triggered [8,9]. The simulated position was gradually displaced at a constant deviation rate α, reproducing the induced-spoofing model used in the SITL experiments.

The overall trends observed in the indoor spoofing experiments are consistent with the simulation study. Here, we focus on two representative cases under the same slow deviation rate, *α* = 0.5 m/s.

**Figure 11:**
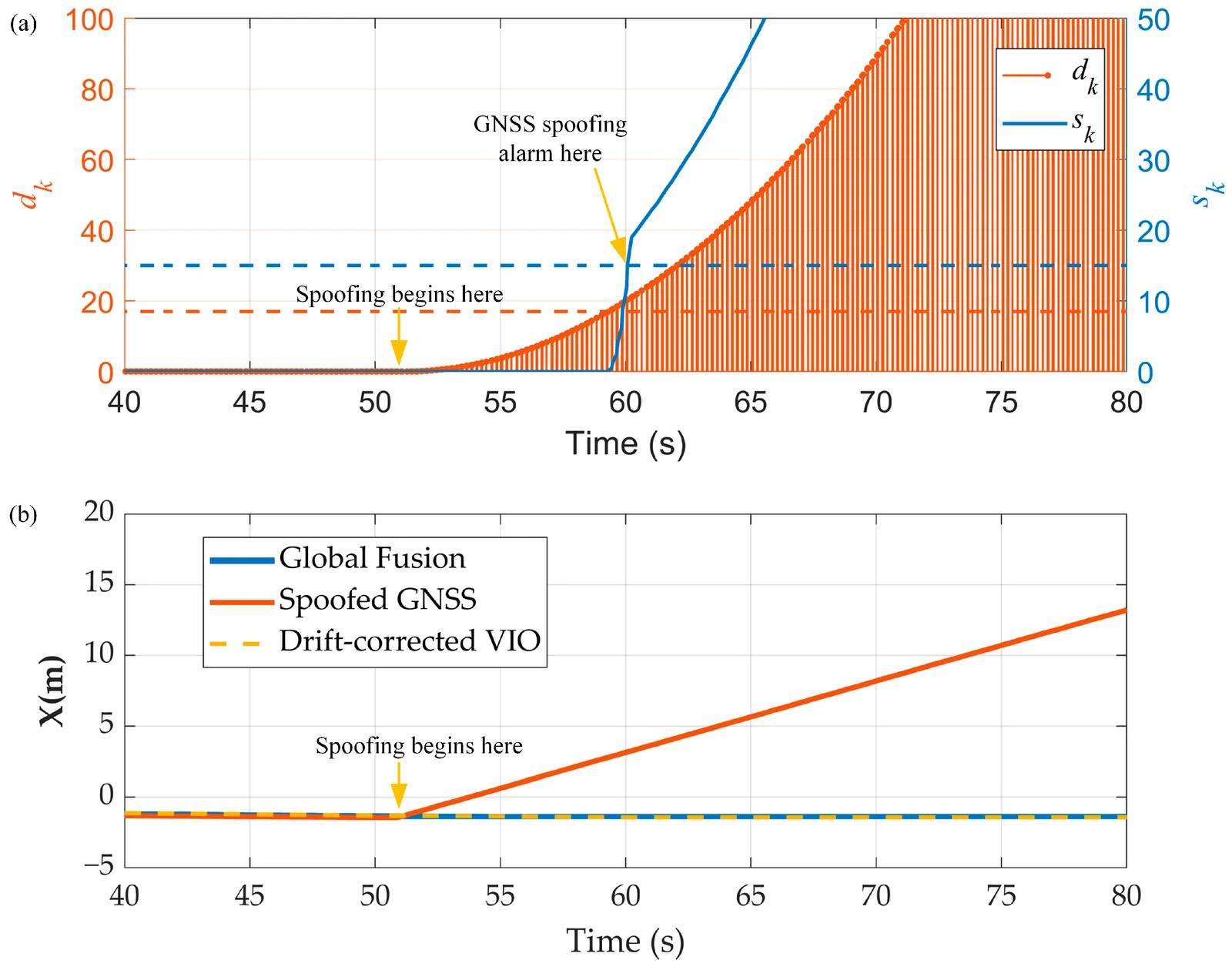
Case 1: successful detection with *t_w_* = 10 s. (**a**) Normalized innovation statistic *d_k_* (left axis) and CUSUM statistic *s_k_* (right axis). The spoofing attack begins at *t* ≈ 51 s and the alarm is raised at *t* ≈ 60 s. The orange and blue dashed lines refer to the parameters *κ* and *γ*, respectively. (**b**) Position estimates along the *X*-axis. The fused solution is not pulled away from the true trajectory.

Case 1: successful detection with *t_w_* = 10 s. The detector was configured with a 10 s time window and thresholds κ = 17, γ = 15. A spoofing attack with deviation rate α = 0.5 m/s was injected at *t* ≈ 51 s. As shown in Figure 11a, the normalized innovation statistic *d_k_* begins to grow once the spoofed solution starts to deviate, and the CUSUM statistic *s_k_* accumulates steadily until it crosses the decision threshold at *t* ≈ 60 s, triggering the spoofing alarm. The refined detection time is recorded at *t* ≈ 59.27 s according to Algorithm 1. Because the buffered GNSS measurements are quarantined rather than fused directly, the VIO drift-correction parameters remain anchored to the healthy measurements acquired before the attack, and the fused position estimate is accordingly not pulled away from the true trajectory, as shown in Figure 11b. The resulting detection latency of approximately 8.27 s is consistent with the mean detection time obtained in simulation for the same configuration (Table 4), confirming that the SITL platform faithfully reproduces the detection behavior of the onboard pipeline.

**Figure 12:**
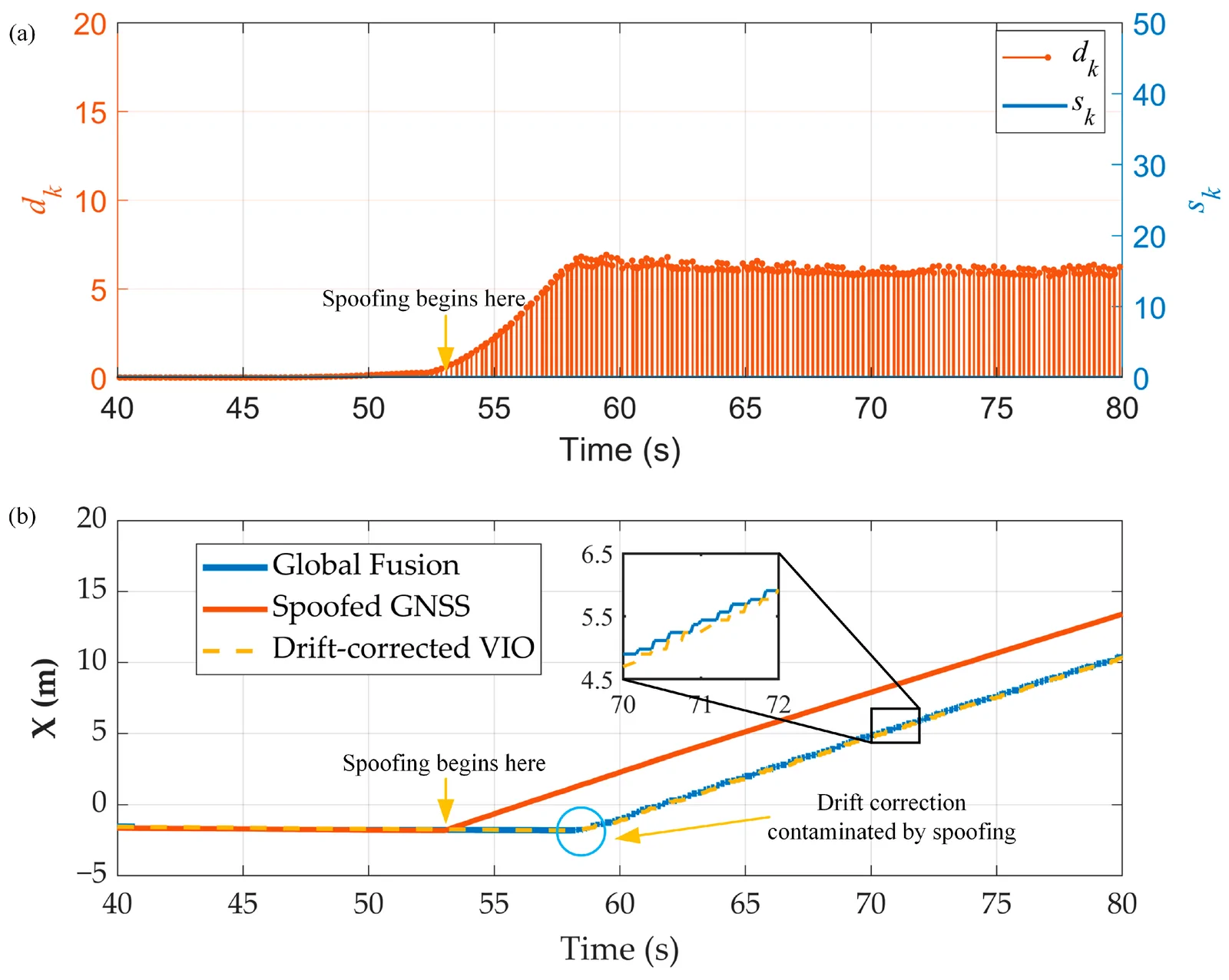
Case 2: missed detection with *t_w_* = 5 s. (**a**) Normalized innovation statistic *d_k_* (left axis) and CUSUM statistic *s_k_* (right axis). *d_k_* peaks at *t* ≈ 58 s, but *s_k_* remains below the decision threshold γ, so no alarm is raised. (**b**) Position estimates along the *X*-axis. The fused solution is progressively pulled toward the spoofed trajectory.

Case 2: missed detection with *t_w_* = 5 s. With a shorter 5 s time window and the corresponding thresholds κ = 14 and γ = 15, an attack with the same deviation rate α = 0.5 m/s was injected at *t* ≈ 53 s. As shown in Figure 12a, the normalized innovation statistic *d_k_* reaches its maximum at *t* ≈ 58 s, but the CUSUM statistic *s_k_* remains below γ and no alarm is raised. The underlying mechanism is identical to that identified in the simulation: because the buffered measurements are accepted as trustworthy after *t* ≈ 58 s, the spoofed solution is fused into the global estimator, and the drift-correction module progressively attributes the GNSS–VIO discrepancy to its own drift. The drift-corrected VIO reference is consequently pulled toward the spoofed trajectory, the innovation statistic is suppressed, and the fused position is displaced after *t* ≈ 58 s, as shown in Figure 12b. This case reproduces on the physical platform the stealthy-spoofing failure mode predicted by the SITL evaluation, demonstrating that slowly varying attacks remain intrinsically difficult to detect.

Taken together, the two cases show that the detection-aware delayed mechanism is the decisive design for stealthy spoofing detection: it must be long enough for the spoofing-induced innovation to accumulate faster than the VIO drift over the same interval. The hardware results further demonstrate that the proposed framework can be deployed on a lightweight platform without modifying the detection logic.

## 5. Discussion

A central consideration in auxiliary measurement-assisted GNSS spoofing detection is that available dead racking positioning, such as VIO, cannot serve as a drift-free, statistically independent positioning reference. While VIO provides locally consistent relative motion, its position error accumulates over time, and global-frame alignment generally requires drift estimation and correction using historical GNSS observations. Consequently, the globally aligned VIO estimate may become indirectly coupled with GNSS: if an unverified GNSS measurement updates the drift-correction state, spoofing-induced errors propagate into the VIO reference, reducing the GNSS–VIO discrepancy and weakening subsequent detection. The framework proposed here explicitly accounts for this coupling by decoupling integrity assessment from the VIO drift-correction update. Recent GNSS measurements are quarantined before fusion, and only measurements that pass integrity screening are permitted to update the factor graph and the associated drift correction.

The delayed-fusion mechanism is not intrinsically tied to the CUSUM detector used in the present implementation. CUSUM was selected for its computational efficiency in accumulating small but persistent inconsistencies characteristic of gradual spoofing. Any detector capable of producing an integrity decision could in principle be integrated into the framework. The proposed framework should therefore be understood as a detector-agnostic, integrity-aware fusion architecture, with CUSUM serving as one lightweight realization.

The fusion-window length governs the trade-off between integrity assurance and navigation accuracy. A short window permits valid GNSS measurements to enter the estimator quickly but provides limited evidence for distinguishing a slowly growing spoofing discrepancy from nominal noise and VIO drift. A longer window allows persistent inconsistencies to accumulate but delays the use of valid GNSS information and increases dead-reckoning uncertainty. Moreover, the detection threshold generally must increase with the observation interval to maintain an acceptable false-alarm rate, so extending the window improves sensitivity only within a limited range.

Consequently, slowly varying spoofing remains fundamentally challenging because attack-induced displacement and dead-reckoning drift may exhibit similar temporal signatures. When their growth rates are comparable within the observation interval, a detector based solely on GNSS–VIO consistency has limited statistical power to separate the two processes. Delayed fusion prevents unverified GNSS measurements from immediately contaminating the reference but cannot eliminate the intrinsic uncertainty of VIO or guarantee detection of arbitrarily slow attacks. Additional evidence sources may therefore be needed for demanding scenarios.

Several limitations define the scope of the present results. First, although the proposed framework explicitly models and compensates for accumulated VIO drift, its integrity monitor still assumes that VIO remains sufficiently reliable within the time window. Low-texture or repetitive scenes, poor illumination, motion blur, and rapid rotation may cause abrupt VIO degradation that cannot be captured by a gradual-drift model alone. Such degradation may enlarge nominal residuals, bias the drift-correction estimate, or be misinterpreted as GNSS spoofing. Future extensions should therefore incorporate VIO health monitoring to distinguish GNSS anomalies from failures of the auxiliary reference. Nevertheless, explicitly representing the VIO drift-correction state and protecting it from unverified GNSS updates constitutes an important step beyond treating VIO as an independent, drift-free measurement source.

Second, the present experiments consider constant-rate gradual position displacement. Adaptive adversaries could vary the injection rate in response to the estimated detection state, pause and resume attacks, coordinate position and velocity manipulation, or exploit changes in vehicle motion and sensing conditions. The closed-loop SITL platform provides a suitable basis for extending this analysis, as attack profiles, controller responses, vehicle dynamics, and sensor outputs can be varied systematically and repeatedly without the safety and regulatory constraints of radiated field experiments. It can therefore support large-scale Monte Carlo evaluation of adaptive, intermittent, multi-stage, and motion-dependent attacks before selected cases are transferred to controlled hardware testing. However, a systematic comparison between open-loop injection versus closed-loop SITL requires substantial additional experiments and is deferred to future work.

Third, the hardware experiments were conducted on a stationary indoor platform using a laboratory GNSS signal simulator. This setting isolates the estimation and detection pipeline and avoids uncontrolled radio-frequency radiation, but it does not reproduce the complete navigation–control dynamics feedback loop. Outdoor experiments involving radiated spoofing signals are difficult to conduct because unintended signal leakage may interfere with nearby GNSS receivers and safety-critical services. Such tests require an authorized site, spectrum approval, geographic isolation, power control, monitoring equipment, and procedures to prevent spoofing signals from escaping the designated test area. Consequently, future validation should combine closed-loop SITL analysis, shielded or conducted RF testing, and carefully authorized outdoor flight trials.

## 6. Conclusions

This study addressed GNSS spoofing mitigation for lightweight UAVs from two complementary perspectives: improving the causal fidelity of spoofing-resilience evaluation and preventing unverified GNSS measurements from contaminating the navigation state. A closed-loop software-in-the-loop (SITL) platform was established to couple GNSS spoofing injection with state estimation, flight control, vehicle dynamics, and subsequent sensor observations. The platform captures a feedback pathway that is not necessarily exercised in prescribed-trajectory or offline replay evaluations. Based on this platform, a detection-aware delayed multi-sensor fusion framework was developed by combining a loosely coupled factor-graph estimator with a sliding-window CUSUM detector. Recent GNSS factors are quarantined and incorporated into the factor graph only after integrity screening, thereby reducing the risk that spoofed measurements contaminate the globally aligned VIO estimate and suppress the residual used for detection. Monte Carlo simulations quantified the trade-off among false-alarm rate, detection probability, and latency. Under the evaluated sensor conditions and gradual-drift attack model, attacks with deviation rates of at least 1.0 m/s were detected within approximately 4–5 s, while detecting the slowly deviation rate at 0.5 m/s required a 10–15 s window and achieved a detection probability exceeding 96% with a false-alarm rate of approximately 3%. The implementation consumed less than 10% CPU and approximately 350 MB of memory during a 30 min onboard test, demonstrating real-time feasibility on the evaluated lightweight platform while indicating the need for bounded graph management during longer missions. Controlled indoor GNSS injection experiments reproduced the principal estimation and detection behaviors observed in simulation. The results further show that slowly varying attacks remain difficult to distinguish from accumulated VIO drift. Future work will therefore address VIO degradation, adaptive and intermittent attacks, adaptive time windows, additional GNSS consistency information, fixed-lag graph management, and controlled outdoor flight validation.

## Figures and Tables

**Table 1:** Key parameters for the simulation.

Parameter	Value	Parameter	Value
Max. flight speed	10 m/s	IMU sampling rate	200 Hz
GNSS sampling rate	10 Hz	Accelerometer random walk	$0.2\;m/s/\sqrt{h}$
GNSS position noise (1σ)	1.0 m	Accelerometer-bias standard deviation	250 mGal
Camera sampling rate	20 Hz	Gyroscope random walk	$0.2{}^{\circ}/\sqrt{h}$
Resolution of cameras	640 × 480	Gyroscope-bias standard deviation	10°/h

**Table 2:** Attack-free position-estimation accuracy under different time window length.

*t_w_* (s)	0	1	5	10	15	20	25
RMSE (m)	0.47 ± 0.04 ^1^	0.53 ± 0.07	1.02 ± 0.12	1.40 ± 0.16	1.85 ± 0.21	2.19 ± 0.22	2.46 ± 0.27

^1^ Values represent the mean ± standard deviation of RMSE.

**Table 3:** Parameter selection for the spoofing detector.

Time Window Length (s)	*κ*	*γ*
0	3.5	6
1	6	15
5	14	15
10	17	15
15	25	15
20	35	25
25	35	25

**Table 4:** Spoofing detection performance under varying deviation rates α and time window length *t_w_*.

Time Window (s)	Deviation Rate *α* (m/s)				
	0.5	1.0	1.5	2.0	3.0
1	- ^1^	-	-	5% (1.27 ± 0.43)	100% (0.67 ± 0.09) ^2^
5	-	100% (3.59 ± 0.25)	100% (2.42 ± 0.20)	100% (1.77 ± 0.18)	100% (1.13 ± 0.15)
10	96% (8.62 ± 3.12)	100% (4.05 ± 0.49)	100% (2.56 ± 0.41)	100% (1.75 ± 0.31)	100% (1.07 ± 0.21)
15	100% (10.05 ± 1.91)	100% (4.45 ± 0.93)	100% (2.99 ± 0.58)	100% (2.12 ± 0.45)	100% (1.29 ± 0.36)
20	100% (10.90 ± 2.08)	100% (5.09 ± 0.94)	100% (3.55 ± 0.67)	100% (2.70 ± 0.61)	100% (1.85 ± 0.42)
25	100% (9.58 ± 3.10)	100% (5.44 ± 1.68)	100% (3.39 ± 1.39)	100% (2.86 ± 0.77)	100% (1.78 ± 0.53)

^1^ denotes that no alarm was raised in any of the 100 runs. ^2^ Detection rate is evaluated per complete attack run. Values in parentheses report the mean ± sample standard deviation of the refined detection-time offset over successfully detected runs only.

## Data Availability

The raw data supporting the conclusions of this article will be made available by the authors on request.

## Abbreviations

The following abbreviations are used in this manuscript:

UAV	Unmanned aerial vehicle
GNSS	Global Navigation Satellite System
IMU	Inertial measurement unit
VIO	Visual–inertial odometry
LIO	LiDAR–inertial odometry
LIVO	LiDAR–inertial–visual odometry
FGO	Factor-graph optimization
SITL	Software-in-the-loop
MAP	Maximum a posteriori
iSAM	Incremental smoothing and mapping
CUSUM	Cumulative sum
FAR	False alarm rate
ENU	East-North-Up
ROS	Robot Operating System
